# IL-4R and CXCR2 Contribute to Downregulating Neutrophil-Mediated Response in the Early Stage of Fungal Extract-Induced Allergic Airway Inflammation

**DOI:** 10.3390/biomedicines12122743

**Published:** 2024-11-30

**Authors:** Marina A. Shevchenko, Ekaterina A. Servuli, Dina E. Murova, Julia D. Vavilova, Elena L. Bolkhovitina, Ekaterina N. Chursanova, Alexander M. Sapozhnikov

**Affiliations:** 1 Department of Immunology, Shemyakin and Ovchinnikov Institute of Bioorganic Chemistry, Russian Academy of Sciences, 117997 Moscow, Russia; servuli@yandex.ru (E.A.S.); murovadina7@gmail.com (D.E.M.); juliateterina12@gmail.com (J.D.V.); alenkash83@gmail.com (E.L.B.); eka.bio@mail.ru (E.N.C.); 2 Laboratory of Studies of Bone and Metabolic Effects of Microgravity, Institute of Biomedical Problems, Russian Academy of Sciences, 123007 Moscow, Russia; 3 Department of Molecular Medicine, University of Padua, 35121 Padua, Italy; 4 Department of Biology, Lomonosov Moscow State University, 119991 Moscow, Russia

**Keywords:** allergic airway inflammation, *Aspergillus fumigatus* extract, eosinophils, neutrophils, IL-4, CXCL1, CD124, CXCR2

## Abstract

**Background/Objectives**: Airborne exogenous antigen inhalation can induce neutrophil infiltration of the airways, while eosinophils migrate to the airways in allergic airway inflammation. During a bacterial infection, Th2-associated cytokine IL-4, by binding to the IL-4 receptor (IL-4R), can suppress neutrophil recruitment to the site of inflammation. In the present study, we estimated whether the IL-4-dependent suppression of neutrophil recruitment contributed to the development of an immune response in asthma. **Methods**: Using a mouse model of *Aspergillus fumigatus* extract-induced allergic airway inflammation, we investigated the proportions of eosinophils and neutrophils in blood, lungs, and bone marrow over time. Bronchoalveolar lavage (BAL) fluid cytokine (including IL-4) levels and the proportions of bone marrow IL-4Rα (CD124)-expressing neutrophils were estimated. **Results**: We identified skewing from the neutrophil- to eosinophil-mediated immune response in the blood after five extract applications. At this point, the BAL fluid IL-4 level was not elevated, while IL-12p40 and CXCL1 levels were considerably increased. At the early stage of allergic airway inflammation, the proportions of neutrophils expressing CD124 and circulating neutrophils expressing CXCR2 (CD182) were significantly increased. Upon inflammation progression, the former remained elevated, but the latter significantly decreased. **Conclusions**: Thus, in allergic airway inflammation, bone marrow neutrophils become insensible to the attractive chemokine CXCL1 signals and susceptible to IL-4 effects.

## 1. Introduction

Asthma is a chronic airway disorder with several endotypes characterized by different clinical manifestations [1,2,3,4]. Asthma with fungal sensitization is usually characterized as severe allergic airway inflammation [5]. Experimental mouse models demonstrated elevated neutrophil attraction in response to a single dose of fungal spores, while eosinophil lung infiltration is observed after multiple applications of spores or extracts [5,6,7]. Besides the doses of antigen/pathogen and repetitive exposure, the mouse strain determines the type of inflammation since distinct granulocyte patterns characterize different strains. Thus, BALB/mice were characterized as predisposed to a neutrophil-mediated response compared to C57BL/6 mice [8,9,10,11]. Although BALB/c and C57BL/6 mice are common for airway inflammation investigations, only several studies have reported on the data obtained in CD-1 mice. Moreover, in ovalbumin-induced airway inflammation, a neutrophil-mediated response is reported, while eosinophilia was observed in a combined model of ovalbumin and Asian sand dust [12,13].

Eosinophils are considered to be mainly effector cells in Type 2 (T2)-high asthma [3,4], while neutrophils play an ambiguous role in the pathogenesis of asthma [14,15]. They can exacerbate airway inflammation in neutrophilic asthma [16,17]. At the same time, some evidence shows neutrophils can suppress allergic responses. Particularly, through TGF-β secretion and via an IL-23/IL-17/granulocyte colony-stimulating factor (G-CSF), regulatory axis neutrophils can affect allergen-specific lymphocytes and limit airway eosinophilia [18,19]. However, during allergic airway inflammation, neutrophils are suppressed and replaced with eosinophils [20,21]. Due to the potential anti-allergic activity of neutrophils, the mechanisms implicated in their unresponsiveness in allergic airway inflammation are essential but still not completely understood.

Several data indirectly indicate that the interleukin (IL)-4 signaling-mediated suppression of neutrophil immune responses can be implicated in the pathogenesis of allergic airway inflammation. IL-4, together with IL-5 and IL-13, are Type 2 (T2)-high asthma-associated cytokines that are assumed to provide the main asthma manifestations, such as bronchial hyperreactivity, mucus hyperproduction, immunoglobulin E (IgE) synthesis, and airway eosinophilia [22,23]. In a long course of allergic airway inflammation, due to T-helper 2 (Th2) activation and proliferation, the secretion of IL-4 is significantly elevated [21,22,23]. IL-4 usually acts through its receptors (IL-4Rs): type 1 and type 2 IL-4Rs. Type 2 IL-4R is comprised of two subunits: IL-13Rα1 and IL-4Rα (also known as CD124), and the expression of this receptor has been reported for bone marrow neutrophils [23,24,25,26]. In bacterial infections, Woytschak et al. [27] demonstrated that by binding to the type 2 IL-4R, IL-4 prevented the interaction of G-CSF with its receptor CXCR2 (also known as CD182). Such prevention subsequently suppressed neutrophil recruitment from the bone marrow to the periphery and affected neutrophil functions, such as migratory activity and neutrophil extracellular traps (NETs) formation [11,27]. Moreover, IL-4R signaling affects the neutrophil precursors and facilitates neutrophil apoptosis [28]. The therapeutic blocking of IL-4 signaling in asthmatics and mouse models decreases IL-5 secretion and eosinophil tissue counts [22,29]. However, the implication of IL-4 signaling in neutrophil immune response suppression during allergic airway inflammation has not yet been demonstrated.

The present study focuses on regulating neutrophil-mediated responses in allergic airway inflammation. We checked the kinetics of neutrophil and eosinophil recruitment to the bloodstream and the airways during *Aspergillus-fumigatus*-extract-induced allergic airway inflammation development. We tested the cytokine secretion (with a focus on IL-4) in bronchoalveolar lavage (BAL) fluids of mice at different stages of inflammatory processes. To check the possible effects of IL-4 on neutrophil attraction, we examined the expression of CD124 on bone marrow neutrophils and estimated the proportion of CXCR2-positive circulating neutrophils. We also estimated the effect of allergic airway inflammation on the functional activity of neutrophils, which were selected from bone marrow cells, particularly ATP-induced chemotaxis, N-formyl-methionyl-leucyl-phenylalanine (f-MLP)-induced reactive oxygen species (ROS) production, and phorbol 12-myristate 13-acetate (PMA)-induced extracellular DNA formation.

Our data support the implication of IL-4R signaling and CXCL-1-CXCR2 signaling in the neutrophil-mediated response suppression during the allergic airway inflammation progression.

## 2. Materials and Methods

### 2.1. Animals and Ethics Statement

Outbred ICR CD-1 and inbred C57BL/6 and BALB/c female mice were purchased from the Animal Facility Pushchino (Pushchino, Russia) and Animal Facility Stezar (Vladimir, Russia). During the experiments, mice were housed in the animal facility of the Shemyakin and Ovchinnikov Institute of Bioorganic Chemistry, Russian Academy of Sciences. The study used female mice of 10–12 weeks; the BALB/c and C57BL/6 mice weight was 18–20 g, and the CD-1 mice weight was 20–30 g. All animal experiments were performed in concordance with the Guide for the Care and Use of Laboratory Animals under a protocol approved by the Institutional Animal Care and Use Committee at the Shemyakin and Ovchinnikov Institute of Bioorganic Chemistry, Russian Academy of Sciences (protocol numbers 308/2020 and 382/2024). The animals were given standard food and tap water ad libitum and housed under regular 12 h dark–light cycles at 22 °C.

### 2.2. Induction of Allergic Airway Inflammation

Mice received five or ten o.ph. applications of *A. fumigatus* extract (Greer Laboratories, Lenoir, NC, USA) in a dose of 4 μg/mouse/application with 72 h intervals. The other group received a single o.ph. application of *A. fumigatus* extract in a dose of 40 μg/mouse. Before each extract administration, mice were anesthetized with isoflurane (Karizoo, Barcelona, Spain).

### 2.3. Blood Collection

The peripheral blood (approximately 180 μL) was collected from the tail vein before and 72 h after the first *A. fumigatus* extract application. Then, 72 h after the fifth and tenth extract applications, the mice were euthanized, and blood was collected from the vena cava (not less than 300 μL). For blood cell analysis, approximately 150 µL of blood was collected in the 1.5 mL test tubes containing 50 µL of heparin solution in a concentration of 500 units/mL (VelPharm, Russia). For the estimation of leucocyte numbers, the heparinized blood was diluted 1:3 with 3% acetic acid (Merck, Germany). Cell nuclei were quantified within 30 min using the Goryaev chamber (Minimed, Bryansk, Russia).

For the serum analyses, blood was collected in the 1.5 mL test tube, stored for 30 min at RT, and then centrifuged at c.a. 500 g using CV 1500 (Biosan, Latvia) for 15 min. The aliquots of supernatants were stored at −20 °C until use.

### 2.4. Blood Cell Analysis by Flow Cytometry

The heparinized blood was transferred to the hemolysis buffer (155 mM NH4Cl (Reachem, Russia); 0.1 mM Na_2_EDTA (Sigma-Aldrich, Saint Louis, MO, USA); 10 mM NaHCO3 (Applichem, Darmstadt, Germany); pH 7.3). It was then incubated for 5 min at RT, then washed twice with a phosphate buffer by centrifugation at 370 g for 5 min. The cells were transferred to a buffer containing 1% BSA (Sigma-Aldrich) and 2 mM Na_2_EDTA (Sigma-Aldrich) and diluted in a volume of 30 µL for the subsequent staining. Before staining, the cells were incubated for 15 min with anti-mouse CD16/CD32 (clone 93) (Miltenyi Biotec, Bergisch Gladbach, Germany). The following set of anti-mouse antibodies was used (all from Miltenyi Biotec) in accordance with the manufacturer’s recommendations: Ly6G-VioBlue (clone REA526), CD11b-VioGreen (clone REA592), FcεR1-PE (clone REA1079), SiglecF-PE-Vio615 (clone REA798); CD172-PE-Vio770 (clone REA1201), CD124-APC (clone REA235), CD45-APC-Vio770 (clone REA737). In a set of experiments, CD182-APC-Vio770 (clone REA942) was used. For isotype controls, recombinant human IgG1 antibodies conjugated with the correspondent fluorophore (all from Miltenyi Biotec) were used. The samples were incubated for 30 min at room temperature in a place protected from light. SytoxGreen (Invitrogen, Eugene, OR, USA) in a dilution of 1:1,000,000 was used for live/dead cell detection. Analysis was performed using MACSQuant Analyzer 10 (Miltenyi Biotec). The data were processed using the FlowJo V10.0.7 software (FlowJoEnterprise, Ashland, OR, USA).

### 2.5. Quantitation of Total IgE

The total IgE in the peripheral blood samples was detected using the ELISA MAX Standard Set Mouse IgE kit (BioLegend, San Diego, CA, USA) in accordance with the manufacturer’s recommendations. Sera were used at a 1:100 dilution. Absorbance was measured using a MultiskanFC (ThermoFisher, Waltham, MA, USA) at 492 nm against 690 nm.

### 2.6. Bronchoalveolar Lavage (BAL) Collection

The animals were euthanized, and then a cannula (Hospira, Lake Forest, Il, USA) was inserted into the trachea, and the lungs were washed with 800 µL of DPBS (PanEco, Moscow, Russia) twice. The total BAL volume was 1.3 mL. The samples were centrifuged for 5 min at 500 g. The supernatants were collected, aliquoted, frozen, and stored at −20 °C until use. The pellets were suspended in 1 mL DPBS. The total number of cells was counted using a Goryaev chamber (Minimed). Cells were pelleted using a cytocentrifuge (Shandon Cytospin II, London, UK) for 5 min at 500 rpm. Differential cell staining was performed using Diachem DiffQuick reagents (Abris, St. Petersburg, Russia) in accordance with the manufacturer’s recommendations. For the cytospin differential cell count, at least three non-overlapping fields were analyzed using a light microscope Primo Star (Carl Zeiss, Jena, Germany); no less than 300 cells per sample were estimated.

### 2.7. Cytokine Evaluation

Quantitative analysis of cytokines in BAL fluids of mice was carried out by using Mouse Th Cytokine Panel, Mouse Cytokine Panel 2, and Mouse Macrophage/Microglia Panel (LEGENDplex BioLegend, San Diego, CA, USA) in accordance with the manufacturer’s recommendations. BAL fluid samples were analyzed in a dilution of 1:1. A total of 10,000 events were analyzed at each measurement. Analysis was performed using the MACSQuant Analyzer 10 (Miltenyi Biotec) and LEGENDplex Data Analysis Software Qognit, Version 2024-06-15.

### 2.8. Bone Marrow Cell Extraction and Analysis by Flow Cytometry

The cells were washed from the tibia and femur of mice using 5 mL of DPBS. The bone marrow cell suspension was centrifuged at 300× *g* for 10 min. The pellet was then suspended in a hemolysis buffer in a volume of 3 mL per sample and kept at room temperature for 10 min. Then, 7 mL of DPBS was added, and the cell suspension was centrifuged as indicated above. Then, the cells were washed out twice with 5 mL of DPBS using centrifugation as described above and resuspended in a buffer containing 1% BSA (Sigma-Aldrich) and 2 mM EDTA (Sigma-Aldrich) in a concentration of 10 × 10^6^ cells/mL. An aliquot of 30 µL of the suspension was used for each sample.

The cells were incubated for 15 min with anti-mouse CD16/CD32 (Miltenyi Biotec). The following set of anti-mouse antibodies (Miltenyi Biotec) was used in accordance with the manufacturer’s recommendations: Ly6G-VioBlue, SiglecF-PE-Vio615, CD124-PE (clone REA235), and CD182-APC-Vio770. The following anti-mouse antibodies were used and termed as Lineage (Lin): CD3-APC (clone REA641), CD19-APC (clone 6D5), CD11c-APC (clone N418), NK1.1-APC (clone PK136), and Ter119-APC (clone Ter-119). SytoxGreen (ThermoFisher) was used to identify live/dead cells. For isotype controls, recombinant human IgG1 antibodies (clone REA293) conjugated with the correspondent fluorophore (all from Miltenyi Biotec) were used. Analysis and data processing were performed as described above.

### 2.9. Neutrophil Isolation and Analysis by Flow Cytometry

Neutrophils were isolated from the bone marrow cell suspension by negative selection using magnetic separation. The following reagents and equipment were used (all from Miltenyi Biotec): Neutrophil Isolation Kit (mouse), separator QuadroMACS, and LD Columns. The separation was performed in accordance with the manufacturer’s recommendations.

For the maturation analysis, neutrophils were transferred to a buffer containing 1% BSA (Sigma-Aldrich) and 2 mM EDTA (Sigma-Aldrich), incubated for 15 min with anti-mouse CD16/CD32 (Miltenyi Biotec) and stained for 30 min with anti-mouse antibodies (Miltenyi Biotec): CD101–PE-Vio770 (clone REA301) and CD182-APC-Vio770. SytoxGreen (Invitrogen) was used for live/dead cell detection. For isotype controls, human IgG1-PE-Vio770 (Miltenyi Biotec) and human IgG1-APC-Vio770 (Miltenyi Biotec) were used.

### 2.10. Neutrophil ROS Production Detection

Mouse neutrophils isolated from bone marrow were suspended in HBSS (PanEco) and placed onto the white plates (ThermoFisher) in the concentration of 5 × 10^5^ cell/mL in a volume of 200 μL per well in the presence of 10 μM luminol (Sigma Aldrich, USA) for 10 min at 37 °C. The cells were then stimulated with fMLP (Sigma Aldrich) in the concentration of 10 µM. Luminescence was detected using GLOMAX Multidetection System (Promega, Madison, WI, USA) in relative units. Data are shown as indexes: the ratio of the maximum luminescence to the base level (before fMLP application).

### 2.11. Detection of NET Formation or Necrosis

Extracellular nucleotides were detected, as it was described by Barrientos et al. [30]. Neutrophils were placed onto the black 96-well plate (SPL Life sciences, Pocheon, Korea) in the concentration of 1 × 10^6^ cells/mL and a total volume of 200 μL per well in the presence of 5 μM SYTOX Green Nucleic Acid Stain (ThermoFisher). The cells were incubated for 4 h (37 °C, 5% CO_2_) in the presence of 20 µM PMA. Fluorescence was detected using the GloMax-Multi Detection System (Promega). The level of fluorescence was detected in relative fluorescence units. Data are shown as indexes: the ratio of luminescence with and without PMA at the same time point after the PMA application. For imaging, neutrophils were incubated in the presence of PMA (as described above) in the cover-glass-bottom slide (ibidi, Graefelfing, Germany). The cells were fixed with 4% paraformaldehyde (15 min, room temperature). The cells were washed three times and stained with a rabbit polyclonal anti-Histone H3 (citrulline R2 + R8 + R17) antibody (Abcam, Boston, MA, USA) (2 h, room temperature). The cells were washed as described above and stained with donkey-anti-rabbit-DyLight649 antibodies (clone Poly4064) (BioLegend) for 1 h at room temperature in the dark. The cells were washed three times as described above and counterstained with Hoechst 33342 (ThermoFisher). Images of PMA-treated neutrophils were obtained by confocal laser scanning microscopy.

### 2.12. Neutrophil Chemotactic Activity Estimation

The chemotactic activity was detected based on the in vivo migration assay protocol [31]. Neutrophils were transferred in RPMI-1640 (PanEco) and placed in the upper chamber of Transwell (Costar, Glendale, AZ, USA) in the concentration of 1 × 10^6^ cells/mL. Migration was induced by adding ATP dissolved in the concentration of 1 µM to the lower chamber. Then, 1.5 h later, the cells from the lower chamber were quantified using a Goryaev chamber (Minimed). The results are shown as a chemotactic index, calculated as the number of cells in the lower chamber containing ATP divided by the number of cells in the chamber containing the medium alone.

### 2.13. Whole-Mount Conducting Airway Specimen Preparation and Staining

The lungs of the mice were inflated-fixed with 2% paraformaldehyde and stored at +4 °C overnight. The main axial airways of the left and right lower lobes were cut out using a stereomicroscope (Carl Zeiss) and micro-scissors. The airway specimens were then 2 × 1 h washed with DPBS using an orbital shaker (ApexLab, Moscow, Russia) 150 rpm, permeabilized with 0.3% Triton X-100 diluted in DPBS (T-DPBS), and then washed 3 times for 10 min in DPBS. Nonspecific antibody binding was blocked with 1% BSA-DPBS. Samples were stained by incubation with anti-mouse SiglecF-APC (clone S17007L, dilution 1:50) (BioLegend) and anti-mouse Ly6G-AlexaFluor488 (clone 1A8, dilution 1:50) (BioLegend) overnight at +4 °C. After washing, as described previously, the samples were stained with Phalloidin-Atto425 (Sigma-Aldrich, St. Louis, MO, USA ) in dilution 1:50. For isotype control staining, rat IgG2a-AlexaFluor488 and rat IgG2a-APC conjugated (BioLegend, dilution 1:50) were used. All samples were covered with Prolong Gold mounting medium (Thermo Fisher, P36930) and stored at −20 °C until use.

### 2.14. Confocal Laser Scanning Microscopy

Three-dimensional images were obtained using an inverted confocal laser scanning microscope Ziess LSM980 (Carl Zeiss, Jena, Germany). A 63x objective (NA = 1.4 with oil immersion) was used to image the region of interest. The following lasers were used: 405, 488, and 639 nm. The fluorescence was detected using a 34-channel QUASAR detector (Carl Zeiss) tuned to a 405-695 nm range. Spectral unmixing was performed using the ZEN 2.5 (Carl Zeiss) software. The image processing was performed using Imaris 9.8 (Oxford Instruments, GB). The final image processing was performed using Adobe Photoshop CS version 5 (Adobe Systems, Mountain View, CA, USA).

### 2.15. Statistical Analysis

Statistical analysis was carried out using the GraphPad Prism 6 software (GraphPad Software, San Diego, CA, USA). The charts show medians and interquartile ranges. The data were analyzed using the Mann–Whitney U-test. The difference in values at *p* ≤ 0.05 was considered significant.

## 3. Results

### 3.1. CD-1 Outbred Mice Are Prone to Eosinophil-Mediated Immune Response

Previous investigations of *A. fumigatus* conidia-induced allergic responses demonstrated distinct inflammation profiles in the airways of BALB/c and C57BL/6 mice: neutrophils predominated in BALs of BALB/c, while eosinophils represented the majority of BAL cells in C57BL/6 mice [8]. In this study, we investigated the prevalence of neutrophils and eosinophils in the blood of intact inbred BALB/c and C57BL/6 mice. We also included outbred CD-1 mice since the ability for neutrophilic and eosinophilic airway inflammation was reported for this strain [12,13].

**Figure 1:**
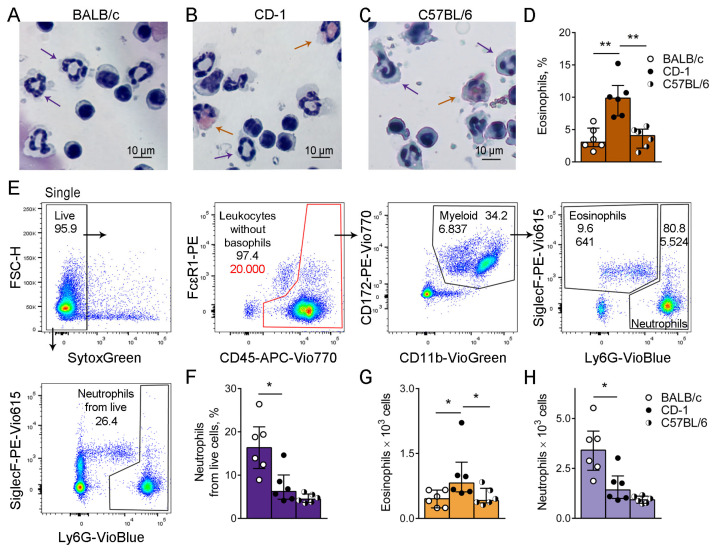
The proportions of blood eosinophils and neutrophils in different strains of mice. (**A**–**C**). Representative images of cytospin blood samples of intact mice BALB/c (**A**), CD-1 (**B**), and C57BL/6 (**C**). Orange arrows indicate eosinophils and violet arrows indicate neutrophils. Scale bar 10 µm. (**D**). Differential eosinophil counts in blood samples of intact mice BALB/c (empty circles), CD-1 (black circles), and C57BL/6 (half-black circles) are demonstrated in percentages. Data are shown as median and IQR (n = 6 mice per group); **: *p* ≤ 0.01. (**E**). Representative dot-plots demonstrating the gating strategy for determination of eosinophils and neutrophils from peripheral blood myeloid cells (upper panel) and neutrophils from whole peripheral blood live cells (low dot-plot) in a sample collected from the BALB/c intact mouse. (**F**). The proportions of neutrophils from live blood cells. (**G**,**H**). The numbers of eosinophils (**G**), and neutrophils (**H**) from 20,000 leukocytes (the gate is indicated with red in (**E**). Data are shown as median and IQR (n = 6 mice per group); *: *p* ≤ 0.05.

The percentages of blood eosinophils were initially detected by the cytospin differential cell count (Figure 1A–C). Unexpectedly, we observed a significant elevation of the eosinophil percentage in the blood of CD-1 mice compared to that of BALB/c and C57BL/6 mice (Figure 1D).

Then, using flow cytometry and the strategy recommended by Liu et al. [32] with modifications, we detected eosinophil and neutrophil populations in the peripheral blood myeloid cells (Figure 1E upper panel, Figure S1) and neutrophils in live blood cells (Figure 1E, low dot plot, Figure S1). The proportion of neutrophils from live blood cells was elevated in BALB/c compared to C57BL/6 and CD-1 mice (Figure 1F). The observation was in line with the report by Fei et al. [8] on the predominance of neutrophils in BALs of BALB/c mice compared to C57BL/6, supporting the predisposition of BALB/c to the neutrophil-mediated response.

Considering the significant difference in total leucocyte numbers in the peripheral blood of CD-1 mice compared to BALB/c and C57BL/6, we estimated the numbers of neutrophils and eosinophils. For this, we limited the gate of leukocytes to 20,000 events (Figure 1E, the gate is indicated with red). We observed that the number of eosinophils was significantly higher in the blood of CD-1 mice (Figure 1G), and the number of neutrophils was significantly elevated in the blood of BALB/c (Figure 1H).

Thus, according to the immune profiles of blood myeloid cells in intact mice, CD-1 mice were predisposed to the eosinophil-mediated immune response, while BALB/c to the neutrophil-mediated one.

### 3.2. Eosinophils Replaced Neutrophils in the Peripheral Blood of Mice with Allergic Airway Inflammation

**Figure 2:**
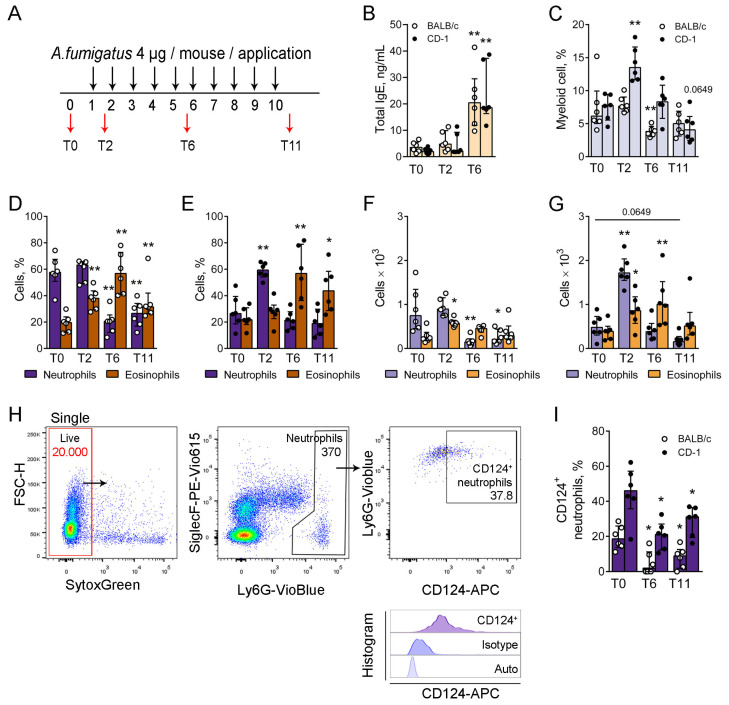
The peripheral blood neutrophil and eosinophil kinetics during allergic airway inflammation. (**A**). The scheme of allergic airway inflammation induction via o.ph. applications of *A. fumigatus* extract (black arrows). Blood sample collections (red arrows) were performed before the first application (T0), 72 h after the first application (T2), 72 h after the fifth application (T6), and 72 h after the tenth application (T11). (**B**). Total serum IgE level in ng/mL, serum dilution 1:100. (**C**). The proportions of myeloid cells from the peripheral blood leukocytes. (**D**,**E**). The proportions of eosinophils and neutrophils from the peripheral blood myeloid cells. (**F**,**G**). The numbers of neutrophils and eosinophils from 20,000 leukocytes. (**H**). Representative dot-plots demonstrating the gating strategy for determining of CD124^+^ neutrophils in the blood of CD-1 intact mouse. Histogram (low image) characterizes CD124 recognition. (**I**). The proportions of CD124^+^ neutrophils in the peripheral blood. Data are shown for BALB/c (white circles) and CD-1 (black circles) mice at different time points (T0, T6, and T11) of induced allergic airway inflammation. Data are shown as median and IQR (n = 6 mice per group). Significant difference between T0 and the indicated time point is *: *p* ≤ 0.05, **: *p* ≤ 0.01.

To check whether the immune profile of blood myeloid cells can affect the immune response to the inhaled allergen, we induced allergic airway inflammation in BALB/c and CD-1 mice by multiple oropharyngeal (o.ph.) applications of *A. fumigatus* extract (Figure 2A). The model was developed based on the well-established model of allergic airway disease in response to house dust mites [18,20]. Leukocyte kinetic analysis in murine BAL fluid and conducting airway mucosa after the allergen challenge served as a rationale for choosing the time point 72 h after the last application for the analysis [21,33].

We estimated the peripheral blood serum IgE level as the standard marker of allergic reaction. A single low-dose allergen application was not potent enough to elevate the total IgE level significantly, while 72 h after the fifth allergen application (T6), the level of total IgE was significantly elevated compared to that of intact mice (Figure 2B).

Using flow cytometry and the gating strategy described above, we compared the proportions of myeloid cells from peripheral blood leukocytes (Figure 2C) and the proportions of neutrophils and eosinophils from these myeloid cells (Figure 2D,E). The myeloid cell proportion was elevated significantly 72 h after the first extract application (T2) in CD-1 but not in BALB/c mice (Figure 2C). The elevation was caused mainly by neutrophil recruitment, as 72 h after the first allergen application, in CD-1 mice, the proportion of neutrophils was significantly elevated compared to intact mice (Figure 2E). Further, 72 h after the fifth (T6) and tenth (T11) application, the proportions of myeloid cells were not significantly different from intact mice in CD-1 and even significantly decreased in BALB/c (Figure 2C), similar to the neutrophil proportions (Figure 2D,E). In line with the elevation of serum IgE, the eosinophil proportion elevated significantly at T6 after the fifth extract application and stayed elevated after the tenth (T11) application, both in BALB/c and CD-1 mice (Figure 2D,E).

To check whether the decrease in the peripheral blood neutrophil proportion during allergic airway inflammation progression is caused by eosinophil infiltration or the exhaustion of the neutrophil population, we compared the alterations of neutrophil and eosinophil numbers. For this, we limited the gate of leukocytes to 20,000 events, as described above (Figure 1E, the gate is indicated with red), and compared the absolute numbers of blood neutrophils at the different time points (Figure 2E,F). The analysis revealed the exhaustion of the blood neutrophil population in BALB/c mice (Figure 2F) and the tendency of exhaustion in CD-1 mice (Figure 2F).

We investigated the alteration of CXCR2 and CD124 expression by blood neutrophils in line with the progression of allergic airway inflammation. As expected, most blood neutrophils were CXCR2^+^ (Figure S2). Interestingly, the proportion of CD124^+^ peripheral blood neutrophils decreased in mice with induced allergic airway inflammation (T6 and T11) compared to intact mice in BALB/c and CD-1 mice (Figure 2H,I and Figure S2).

Thus, single allergen application induced neutrophil recruitment to the blood of eosinophil-mediated response-prone CD-1 mice, but not in the case of BALB/c mice with blood neutrophil domination. However, multiple allergen extract applications induced the suppression of neutrophil-mediated immune responses and eosinophil predomination in the peripheral blood of both neutrophil-mediated response-prone BALB/c mice and eosinophil-mediated response-prone CD-1 mice. Moreover, in allergic mice, neutrophils were characterized by a decreased expression of CD124 compared to intact mice.

### 3.3. Neutrophils Did Not Migrate to BAL and Lung Tissues During Progression of A. fumigatus-Induced Allergic Airway Inflammation

**Figure 3:**
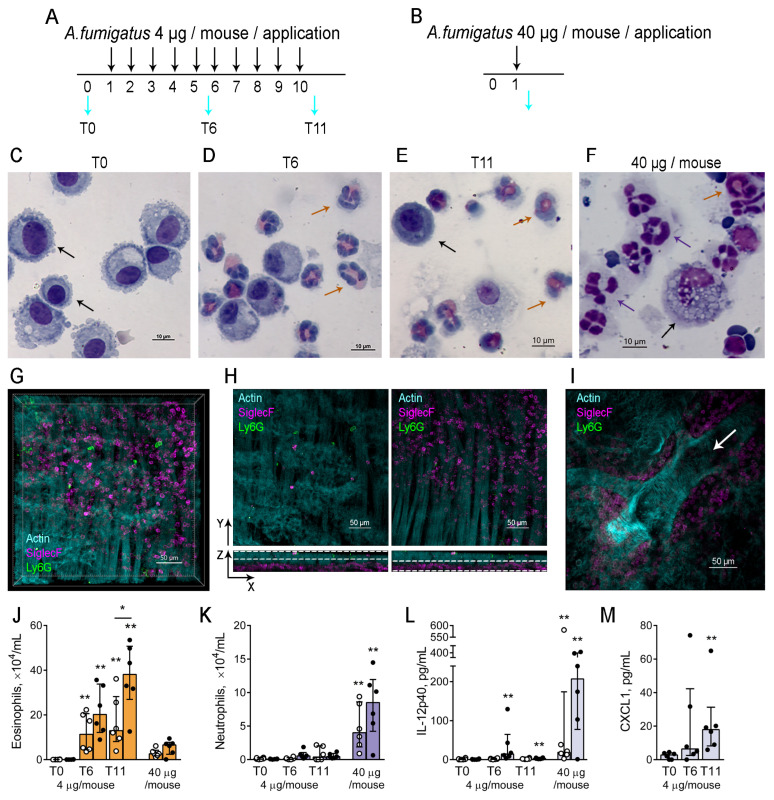
Cellular and humoral response to *A. fumigatus* extract in the airways. (**A**,**B**). The schemes of induction of allergic airway inflammation (**A**) or acute inflammation (**B**). *A. fumigatus* extract o.ph. applications are indicated with black arrows; BAL collections (cyan arrows) were performed before the extract applications (T0), 72 h after the fifth (T6), and 72 h after the tenth (T11) low-dose extract application (**A**), or 72 h after a single high-dose extract application (**B**). (**C**–**F**). Representative images of BAL cytospin samples of intact CD-1 mouse (**C**), CD-1 mice with induced allergic airway inflammation T6 (**D**), T11 (**E**), and CD-1 mouse that received single high-dose extract application (**F**). Black arrows indicate macrophages, violet arrows—neutrophils, and orange arrows—eosinophils. Scale bar 10 µm. (**G**,**H**). Representative image of conducting airway mucosa (**G**,**H**) (**left**) and submucosa (**G**,**H**) (**right**) of CD-1 mouse with allergic airway inflammation at T6 showing eosinophils (SiglecF, magenta), neutrophils (Ly6G, green), and actin filaments of smooth muscle cells (Actin, cyan) as 3D view (**G**) or X-Y-Z projections (**H**). Scale bar 50 µm. (**I**) Representative image of the peribronchial eosinophil infiltration demonstrating as 3D projection. The arrow indicates the direction of the airflow upon inspiration. Scale bar 50 µm. (**J**,**K**). Eosinophil (**J**) and neutrophil (**K**) cell counts in BALs of BALB/c (white circles) and CD-1 (black circles) mice at different time points of allergic airway inflammation (4 µg/mouse) and in mice with acute inflammation (40 µg/mouse). (**L**). BAL IL-12p40 level of BALB/c (white circles) and CD-1 (black circles) mice at different time points of allergic airway inflammation (4 µg/mouse) and in mice with acute inflammation (40 µg/mouse). (**M**). CXCL1 BAL levels of CD-1 mice at different allergic airway inflammation time points. Data are shown as median and IQR (n = 6 mice per group). A significant difference between T0 and the indicated time point or between two indicated time points is shown as * *p* ≤ 0.05, ** *p* ≤ 0.01.

Then, we examined the influence of exposure to an *A. fumigatus* extract on neutrophil recruitment to the airways. Using the scheme of allergic airway inflammation induction described above (Figure 3A) and the control group that received a single application of a high dose of *A. fumigatus* extract (Figure 3B), we estimated the numbers of BAL neutrophils and eosinophils (Figure 3C–F). Additionally, we examined the presence of neutrophils and eosinophils in the lung tissues (Figure 3G–I).

Similar to the decrease in the peripheral blood neutrophil proportion, we observed the suppression of neutrophil recruitment to the airways. In particular, the BALs of intact mice contained macrophages (Figure 3C), and a single application of *A. fumigatus* extract in a dose of 40 µg induced primary neutrophil infiltration (Figure 3F). At the same time, primarily eosinophils were detected in BALs of mice that received five and ten *A. fumigatus* applications (Figure 3D,E). Thus, 72 h after the fifth extract application, more than 20 × 10^4^/mL eosinophils but less than 2 × 10^4^/mL neutrophils were detected in the BALs of CD-1 mice (Figure 3J,K). The same kinetics were observed in the BALs of BALB/c mice (Figure 3J,K). In comparison, 72 h after the single application of a high dose of *A. fumigatus* extract, we detected up to 15 × 10^4^/mL neutrophils in BALB/c mice and up to 15 × 10^4^/mL of neutrophils in CD-1 mice (Figure 3J,K).

In the lung tissue of mice with induced allergic airway inflammation, a few neutrophils and eosinophils were detected in conducting airway mucosa, mainly on the luminal side of the epithelium (Figure 3G,H left image). In contrast, many eosinophils were detected in the submucosal compartment (Figure 3G,H right image). Analysis of the area beneath the smooth muscles bordering the mucosa of the main bronchus revealed that eosinophils located in the peribronchial area of the airways, which were branching from the main bronchus (Figure 3I and Figure S3). However, only a few neutrophils were located in the same region (Figure 3I and Figure S3).

The cytokine production analysis revealed a significant elevation of IL-12p40 in BAL fluids at both T6 and T11 of CD-1, but not BALB/c mice compared to intact mice (Figure 3L). At the same time, the level of IL12-p40 in both CD-1 and BALB/c mice that received a single high dose of extract was significantly higher than that of allergic mice (Figure 3L). Interestingly, we detected significantly elevated levels of CXCL1 in the BAL fluids of allergic CD-1 mice (at T11) compared to intact mice (Figure 3M). Notably, we did not observe the elevation of Th2-associated cytokines in response to five and ten *A. fumigatus* extract applications to BALB/c and CD-1 mice (Supplementary Table S1). We did not observe the detectable levels of IL-12p70, IL-17A, IL-17F, and IL-23 in the BALs of mice with induced allergic inflammation as well as in the mice that received a single high-dose extract application (Supplementary Tables S2 and S3).

Suppressed neutrophil infiltration to the airways was observed in BALB/c and CD-1 mice in response to multiple allergen extract applications. The elevated levels of IL-12p40 and potential neutrophil attractant CXCL1 were detected in the airways of CD-1 mice; however, neutrophils were not recruited in response to these stimuli.

### 3.4. Circulating Neutrophil Numbers Are Altered in the Bone Marrow of Mice with Allergic Airway Inflammation

**Figure 4:**
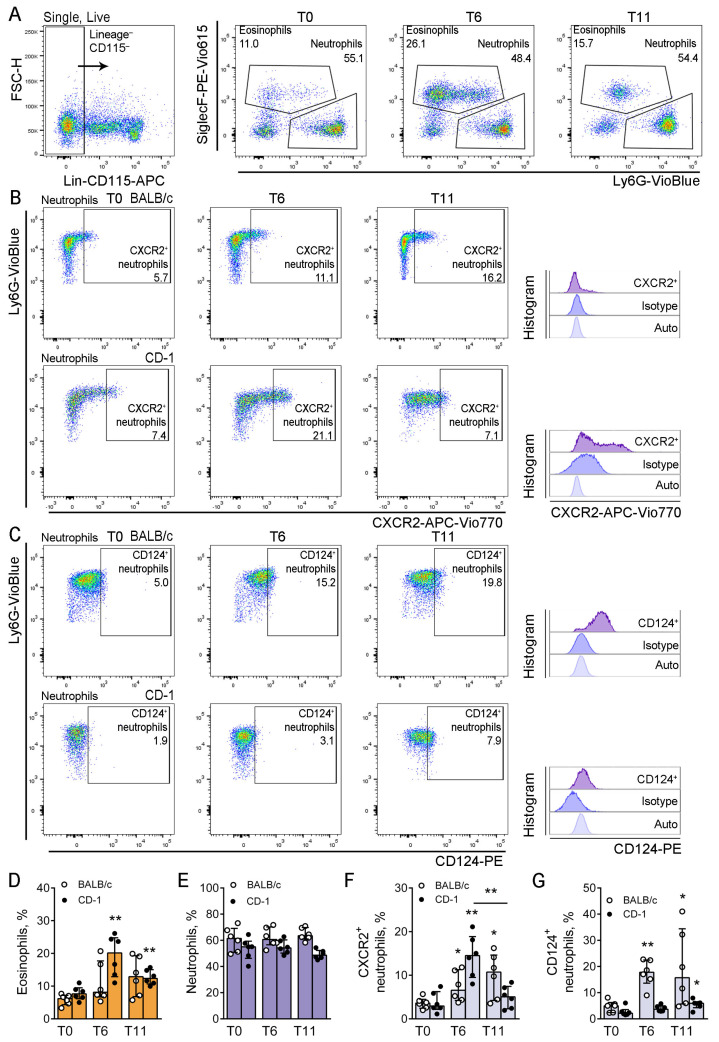
Neutrophil maturation during allergic airway inflammation development. (**A**). Representative dot-plots demonstrating bone marrow neutrophils and eosinophils of CD-1 mice at T0, T6, and T11 of allergic airway inflammation. (**B**,**C**). Representative dot-plots demonstrating CXCR2^+^ (**B**) and CD124^+^ (**C**) bone marrow neutrophils of BALB/c (**upper row**) and CD-1 (**lower row**) mice at T0, T6, and T11. Histograms (**right**) demonstrate isotype-control staining and autofluorescence. (**D**,**E**). The proportions of eosinophils (**D**) and neutrophils (**E**) in the bone marrow of BALB/c (white circles) and CD-1 (black circles) mice at T0, T6, and T11. (**F**,**G**). The proportions of CXCR2^+^ (**F**) and CD124^+^ (**G**) neutrophils from bone marrow neutrophils of BALB/c (white circles) and CD-1 (black circles) mice at T0, T6, and T11. Data are shown as median and IQR (n = 6 mice per group). Significant differences between T0 and the indicated time point or (if indicated) between different time points are shown as * *p* ≤ 0.05, **: *p* ≤ 0.01.

To investigate why neutrophils did not react to the elevated level of CXCL1, we checked the expression of CXCR2 (the marker of bone marrow neutrophils that are ready for the egress to the bloodstream) on bone marrow neutrophils of mice with induced allergic airway inflammation. To discriminate neutrophils among the bone marrow cells, we used the strategy reported previously with modifications [34,35] (Figure 4A). The proportions of CXCR2^+^ bone marrow neutrophils were detected at T0, T6, and T11 (Figure 4B). Since we observed the decrease in CD124^+^ in the blood of mice with allergic airway inflammation, we also detected the proportion of CD124^+^ neutrophils among bone marrow neutrophils at different time points (Figure 4C).

The proportion of eosinophils in the bone marrow significantly increased in mice that received both five and ten *A. fumigatus* extract applications compared to intact mice (Figure 4D). In contrast, the neutrophil proportion did not alter significantly (Figure 4E).

The proportions of CXCR2-expressing bone marrow neutrophils were significantly increased at T6 in both CD-1 and BALB/c mice compared to intact mice (Figure 4F). At T11, the proportion of CXCR2^+^ bone marrow neutrophils was still elevated in BALB/c but not in CD-1 mice; moreover, a significant decrease was observed in CD-1 mice at T11 compared to T6 (Figure 4F).

Less than 10% of bone marrow neutrophils were expressed CD124 in intact BALB/c and CD-1 mice. However, the portions of CD124^+^ neutrophils were significantly increased in the bone marrow of allergic BALB/c mice (at both T6 and T11) compared to that of intact mice (Figure 4G), while in CD-1 mice, the significant elevation in the proportion of CD124^+^ neutrophils was observed at T11 only (Figure 4G).

Thus, the CD124-expressing bone marrow neutrophil population steadily increased in line with allergic airway inflammation progression. In BALB/c mice, the proportion of CXCR2-expressing neutrophils also steadily increased, while in CD-1 mice, the initial elevation skewed to a decrease. That was especially interesting in the context of the elevation of CXCL-1 in the airways of CD-1 mice with induced allergic airway inflammation.

### 3.5. Bone Marrow Neutrophils from Mice with Allergic Airway Inflammation Possess Elevated Motility but Decreased Ability for ROS Production

**Figure 5:**
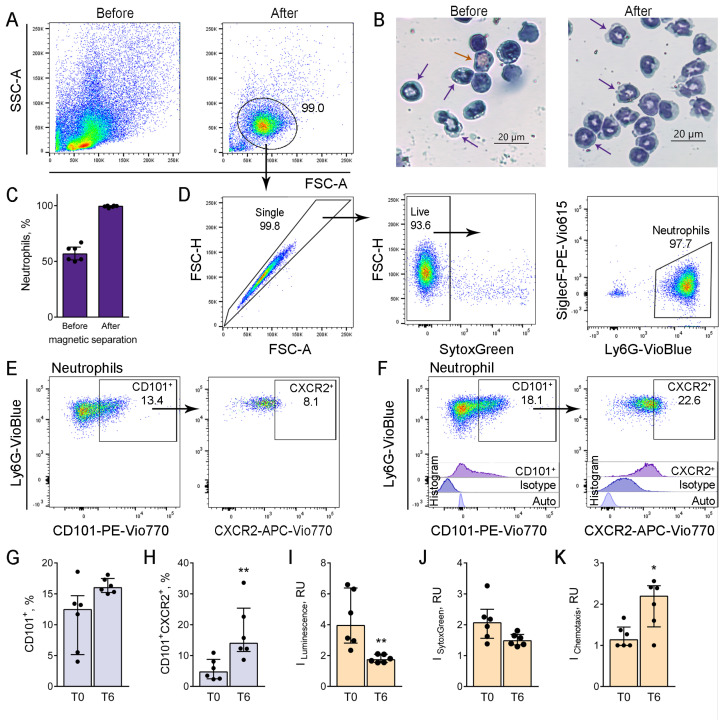
Maturation and functional alterations of bone marrow-derived neutrophils of allergic mice. (**A**). Representative dot-plots demonstrating bone marrow cells of CD-1 mouse with induced allergic airway inflammation (T6) before (**left**) and neutrophils (**right**) after the magnetic separation. (**B**). Representative images of bone marrow cytospin samples of CD-1 mouse with induced allergic airway inflammation (T6) before (**left**) and after (**right**) the magnetic separation. Scale bar 20 µm. (**C**). The proportions of neutrophils in bone marrow cells before and after the magnetic separation according to cytospin counts. (**D**). Representative dot-plots demonstrating neutrophils after magnetic separation of the bone marrow of CD-1 mouse with allergic airway inflammation (T6). (**E**,**F**). Representative dot-plots of CD101^+^ and CD101^+^CXCR2^+^ neutrophils, selected from the bone marrow of intact CD-1 mouse (**E**) and CD-1 mouse with induced allergic airway inflammation at T6 (**F**). (**G**,**H**). The proportions of CD101^+^ neutrophils (**G**) and CXCR2^+^ cells from CD101^+^ neutrophils (**H**) of CD-1 intact mice (T0) and mice with allergic airway inflammation (T6). (**I**). fMLP-induced ROS production is shown as an Index of luminol-dependent chemiluminescence of neutrophils selected from the bone marrow of intact CD-1 mice (T0) and CD-1 mice with induced allergic airway inflammation (T6). (**J**). PMA-induced extracellular nucleotide secretion by neutrophils selected from the bone marrow of intact CD-1 mice (T0) and CD-1 mice with induced allergic airway inflammation (T6). (**K**). Chemotaxis indexes showing migration activities of neutrophils yielded from the bone marrow of CD-1 mice at T0 and T6 in response to 1 µm ATP. Data are shown as median and IQR (n = 4 mice per group). Significant differences between T0 and T6 are indicated as * *p* ≤ 0.05, ** *p* ≤ 0.01.

Since the downregulation of CXCR2 expression that was observed for bone marrow neutrophils of CD-1 mice could be implicated in neutrophil-mediated response suppression during allergic airway inflammation, we checked the functional alterations of bone marrow neutrophils. To this end, we yielded neutrophils from bone marrow cells using negative selection and magnetic separation. Morphological analysis of neutrophils after the separation was performed using flow cytometry and cytospin specimens (Figure 5A,B). The purity of neutrophils was verified by the differential cell staining of cytospin specimens, and the percentage of neutrophils after the separation exceeded 99% (Figure 5C). The flow cytometry also confirmed the purity of neutrophils after the separation (Figure 5D).

We compared the maturation status of neutrophils selected from the bone marrow of allergic CD-1 mice (T6) and intact mice (T0) in accordance with the expression of CD101, a marker of mature neutrophils [34,36] (Figure 5E,F). At T6, there was no significant difference between the proportions of CD101^+^ neutrophils in allergic and intact mice (Figure 5G). At the same time, the proportion of circulating (CXCR2^+^) neutrophils among mature (CD101^+^) neutrophils was elevated in allergic mice (Figure 5H).

Further, these neutrophils possessed a decreased potential for ROS production compared to neutrophils from intact mice (Figure 5I). No significant difference in the extracellular nucleotide secretion was detected between neutrophils of mice with induced allergic airway inflammation and intact mice (Figure 5J and Figure S4). Interestingly, neutrophils from the bone marrow of mice with induced allergic inflammation showed increased chemotactic activity compared to intact mice (Figure 5K).

Thus, in allergic airway inflammation, an increased proportion of mature neutrophils egress from the bone marrow to the bloodstream; therefore, such neutrophil functions as migratory activity and ROS production are affected.

## 4. Discussion

IL-4 and IL-4R are known to be key players in allergic airway inflammation and asthma development and progression. As far as IL-4Rα contributes to both IL-4 and IL-13 signaling, it is considered to be a prospective target for anti-Th2 cytokine therapy in asthma [22,23,24,25,26,29]. Particularly, dupilumab (a drug whose active component is human IgG4 to IL-4Rα) significantly reduced exacerbation frequency in asthmatic patients. Interestingly, blood eosinophil counts increased, while tissue eosinophil count decreased in patients treated with dupilumab. The mechanism of the protective effects of IL-4 Rα blocking is not completely investigated; one of the phenomenon explanations is the IL-4Rα-dependent VCAM-1 and ICAM-1 expression inhibition that prevents eosinophil recruitment to the site of inflammation [22]. In the present study, we have shown the gradual elevation of the IL-4Rα expression on bone marrow neutrophils upon allergic airway inflammation development, indicating the neutrophils as a potential target of IL-4Rα-mediated signaling blockage.

**Figure 6:**
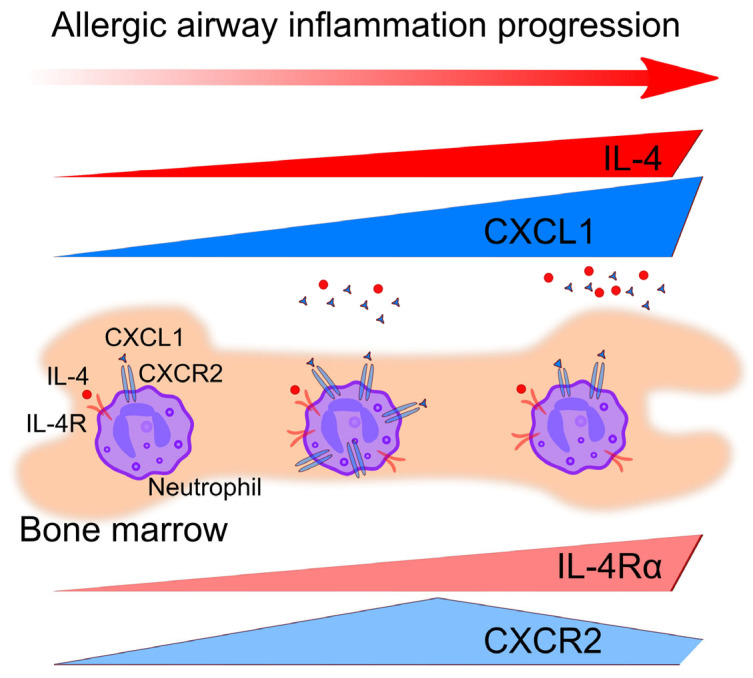
Schematic summary of the implication of IL-4-IL-4Rα and CXCL1-CXCR2 signaling in allergic airway inflammation. During allergic airway inflammation, bone marrow neutrophils differently express two receptors: IL-4Rα (light red) and CXCR2 (light blue). The number of neutrophils that express IL-4Rα—receptor for the pro-allergic cytokine IL-4 (red) elevates at the beginning of allergic airway inflammation and remains elevated further during the inflammation progression. The number of neutrophils expressing CXCR2, whose ligand CXCL1 (blue) is a neutrophil attractant, elevates initially but further decreases. Thus, upon allergic airway inflammation progression, neutrophils become sensitive to the neutrophil suppressor IL-4 but insusceptible to the neutrophil attractant CXCL1.

The efficacy of cytokine therapy in asthma is almost determined by the phenotype of asthma [22,29]. In the present study, we compared two mouse strains: BALB/c with blood neutrophil predominance and CD-1 with eosinophil predominance. Interestingly, in both strains, multiple applications of small-dose *A. fumigatus* extract induced the reduction in the neutrophil blood number. Moreover, despite the elevation of IL-4Rα-expressing neutrophils proportion in the bone marrow, a significant decrease in IL-4Rα-expressing neutrophils in blood was observed in both BALB/c and CD-1 mice. The observation indicates the inability of IL-4Rα-expressing neutrophils to egress from the bone marrow to the bloodstream (Figure 6). Woytschak et al. [27] demonstrated that in the case of local and systemic bacterial infection that induces T2 immune response, IL4-IL-4Rα signaling prevented neutrophil migration from the bone marrow to the bloodstream by blocking the neutrophil-activating signaling through CXCR2. Therefore, we measured CXCR2 expression on neutrophils in mice with induced allergic airway inflammation. In line with previously reported, blood neutrophils were CXCR2-positive primarily [34]. In the bone marrow of BALB/c mice, CXCR2-positive neutrophil proportions were elevated after five and after ten allergen extract applications, while in CD-1 mice, after initial elevation, a significant decrease was observed (Figure 6). That was in line with the significant elevation of IL12p40—so-called “first order cytokine” and CXCL1, which is known as neutrophil attractant CXCR2 ligand, in the BAL fluids of CD-1 mice with induced allergic airway inflammation [37,38]. Notably, no elevated IL-12p40 was observed in BAL fluids of BALB/c mice with allergic airway inflammation; however, in mice that received a single high-dose of *A. fumigatus* extract, IL-12p40 was elevated.

CD-1 mice were previously reported to maintain neutrophil-mediated inflammation and eosinophil-mediated inflammation in different ovalbumin-induced models of airway inflammation induction, obviously due to the heterogeneity of outbred CD-1 mice [12,13]. The eosinophilic phenotype of CD-1 mice was suggested since these mice were traditionally used for obesity mouse models, and eosinophils play an essential role in the link between obesity and asthma [39,40]. Indeed, in the present study, we demonstrated elevated numbers of eosinophils in the peripheral blood of intact CD-1 mice compared to BALB/c and C57BL/6. To prove the blood eosinophilic profile, we used CD-1 mice from different animal facilities, and in some series of experiments, CD-1 and BALB/c mice were held in the same cage. Interestingly, a single low-dose *A. fumigatus* extract application induced the elevation of the neutrophil blood proportion in CD-1 but not BALB/c mice. Further, the neutrophil proportion decreased in line with the elevation of the eosinophil proportion, and 72 h after the fifth allergen application, the proportion of eosinophils significantly increased compared to that of intact mice. We selected this time as the time point of skewing from neutrophil- to eosinophil-mediated immune response. At this point, we observed the character features of allergic airway inflammation as elevated serum total IgE and BAL eosinophilia. Multiple applications of low-dose extract induced eosinophilia in the lung tissues of CD-1 mice; just neglectable neutrophils were observed (mostly on the luminal side of the airway epithelium). The eosinophil-mediated response was observed also in the airways of BALB/c mice. Eosinophils are assumed to be mostly associated with T2-high asthma; however, eosinophilic non-T2 asthma is also reported [3,41]. Although the model we used in the present study is characterized by prominent airway eosinophilia, the levels of Th2 cytokines, particularly IL-4 in the airways of mice, were unexpectedly low, obviously indicating non-T2 allergic airway inflammation.

Previously, we observed massive neutrophil migration in conducting airway mucosa in BALB/c and C57BL/6 mice in response to *A. fumigatus* conidia [6,33]. To prove whether the extract of *A. fumigatus* is also potent in inducing a neutrophil-mediated response in the airways, we applied a single high dose of the extract to mice. We detected a significant elevation of BAL neutrophil percentages in mice that received a single high-dose extract application, while multiple low doses did not activate the neutrophil-mediated response in the airways. These data fall into the concept that neutrophils do not contribute substantially to allergic airway inflammation but are associated with severe or neutrophilic asthma [15,16]. Neutrophil depletion in the mouse model of mixed granulocytic phenotype of allergic airway inflammation exacerbated T2 inflammation and particularly eosinophilia in the airways of mice [18]. In particular, due to the absence of apoptotic neutrophil efferocytosis by macrophages, IL-23/IL-17/granulocyte colony-stimulating factor (G-CSF) axis was dysregulated, and a substantial amount of G-CSF remains in the periphery and serves as an attractant for eosinophils [18]. In this study, using CD-1 mice, we selected the mouse model that resembled eosinophilic asthma without neutrophil-depleting antibody injection by choosing the dose of allergen and times of application. Similar to the case of neutrophil depletion, Th17-response was not activated because we did not detect IL-17 cytokines in the BAL fluids of mice.

As described above, we observed that after ten low-dose *A. fumigatus* extract applications in CD-1 mice, neutrophils did not sense CXCL1 upregulation due to decreased CXCR2 expression (Figure 6). At the same time, after five low-dose extract applications, both CXCR2 on bone marrow neutrophils and its ligand CXCL1 in the BAL fluids were elevated, but neutrophils also did not migrate to the airways. To investigate the reasons for neutrophil unresponsiveness, we tested their functional activity and maturation status. In our study, neutrophils from mice receiving five low-dose extract applications demonstrated elevated chemotactic activity in response to ATP, known as a nonspecific chemoattractant [31]. We observed that at this time point, circulating neutrophils demonstrated mature phenotype since the proportion of mature (CD101^+^) neutrophils among circulating (CXCR2^+^) neutrophils was significantly increased in allergic mice compared to intact mice [34,36,42]. NET formation and ROS production are the most important neutrophil functional activities, which permit the prevention of pathogen dissemination. However, NET formation and ROS production can also induce uncontrolled inflammation and, by forming neutrophil-platelet aggregates, can be involved in the induction of acute lung injury [43,44,45,46]. Moreover, upon netosis and necrosis, neutrophils appear to be undetectable by flow cytometry and not visible as Ly6G-positive cells by confocal laser scanning microscopy, which could explain the absence of neutrophils in the airways of CD-1 mice with induced allergic airway inflammation. Therefore, we checked the susceptibility of neutrophils selected from the bone marrow of CD-1 mice that received five allergen applications to netosis or necrosis; however, no significant difference compared to intact mice was observed. At the same time, ROS production was suppressed. The suppression of neutrophil functional activity was reported as a result of IL-4R engagement in human and mouse neutrophils [11,28]. In CD-1 mice, after five allergen applications, around 20% of IL-4Rα-expressing neutrophils were observed in blood and less than 10% in the bone marrow. Thus, the mature status of neutrophils rather than IL-4-IL4R signaling was probably implicated in the neutrophil functional activity suppression.

In summary, at the early stage of allergic airway inflammation, upon exposure to low doses of the allergen, the IL-4Rα-positive neutrophil proportion elevates in the bone marrow but decreases in the blood. Further allergen exposure decreases the proportion of circulating bone marrow neutrophils (ready to egress from the bone marrow to the bloodstream and further to the inflamed tissue). Thus, we have shown the potential susceptibility of bone marrow neutrophils to IL-4 signaling using a mouse model of allergic airway inflammation. Unexpectedly, we did not achieve elevated levels of Th2 cytokines, particularly IL-4, obviously because we focused on the early stage of the inflammatory process, and in progression, IL-4 level could elevate [20]. The other reason is that IL-4 can be bound by its receptors expressed by the cells rather than neutrophils and, therefore, be poorly detectable in BALs of mice with induced allergic airway inflammation. Further study using intracellular IL-4 analysis will shed light on the presence of this cytokine in the airways. Nevertheless, the susceptibility of neutrophils for signaling through IL-4Rα should be taken in consideration in asthma therapy development. The other essential point is the implication of CXCL1-CXCR2 signaling-mediated neutrophil suppression in allergic airway inflammation that should be further investigated during the development of CXCR1/CXCR2 inhibition therapy for asthma treatment [47,48].

## 5. Conclusions

With the increase in allergen inhalations, the number of neutrophils expressing IL-4Rα in the bone marrow increases, making neutrophils susceptible to IL-4 and/or IL-13 signaling. At the same time, a decrease in bone marrow circulating neutrophil number indicates the suppression of neutrophil recruitment from the bone marrow to the blood. Moreover, the minor number of neutrophils in the lungs indicates the suppression of neutrophil attraction to the site of inflammation. Thus, our data support the evidence of the implication of signaling through IL-4Ra and CXCR2 in neutrophil immune response suppression during allergic airway inflammation.

## Data Availability

The original contributions presented in this study are included in the article/supplementary material. Further inquiries can be directed to the corresponding author(s).

## Supplementary Materials

The following supporting information can be downloaded at https://www.mdpi.com/article/10.3390/biomedicines12122743/s1, Figure S1: Neutrophil detection in the peripheral blood of mice. Figure S2: CXCR2 and CD124 expression by blood neutrophils; Figure S3: Eosinophils and neutrophils in the lung tissue. Figure S4: Imaging of NET formation or necrosis. Table S1: Mouse BAL Th2 cytokines; Table S2: Mouse BAL cytokines; Table S3: Mouse Th BAL cytokines.
