# Supplementary material for: IL-4R and CXCR2 Contribute to Downregulating Neutrophil-Mediated Response in the Early Stage of Fungal Extract-Induced Allergic Airway Inflammation
Source: Biomedicines. 2024 Nov 30;12(12):2743. doi: 10.3390/biomedicines12122743 (PMC11727082; doi:10.3390/biomedicines12122743)
Supplement: Supplementary file 1 [file biomedicines-12-02743-s001.zip › biomedicines-3319178-supplementary.pdf]

Supplementary Figures

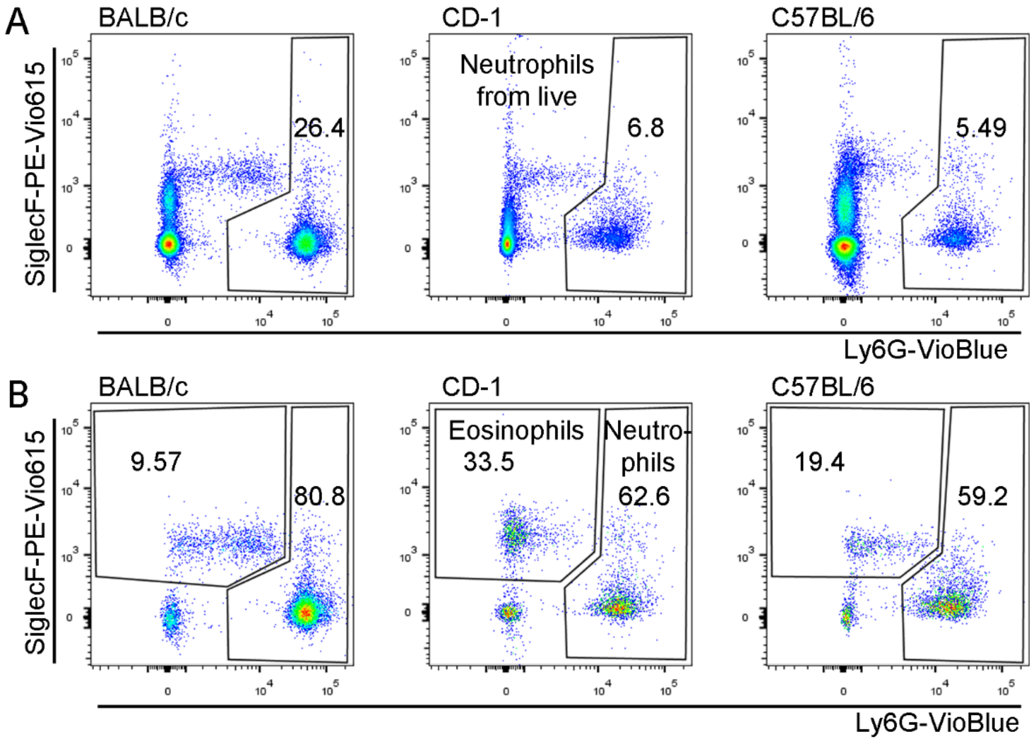

Supplementary Figure 1. Neutrophil detection in the peripheral blood of mice. A, **B**. Representative dot-plots demonstrating neutrophil detection from live blood cells (**A**) and from myeloid cells (**B**) of BALB/c (left plots), CD-1 (middle plots) and C57BL/6 (right plots).

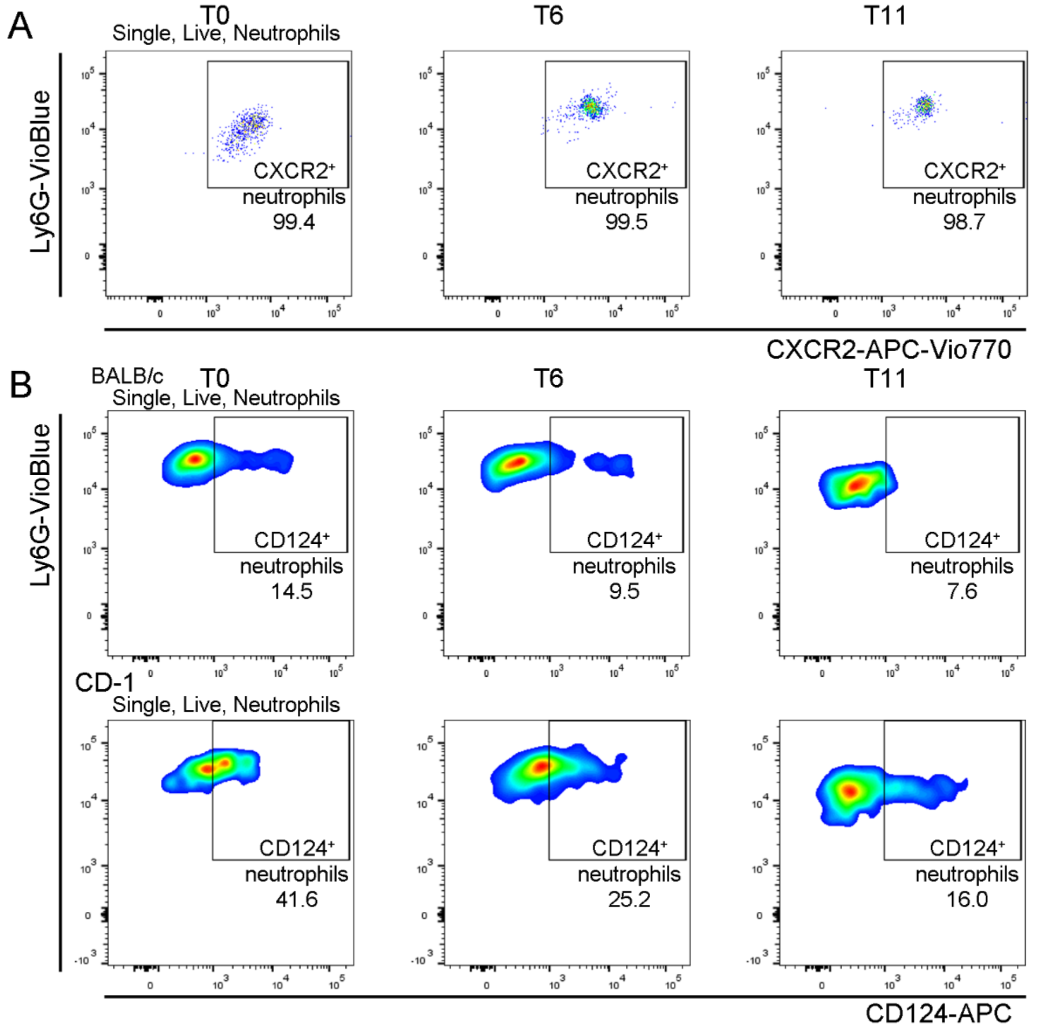

Supplementary Figure 2. CXCR2 and CD124 expression by blood neutrophils. A. Representative dot-plots demonstrating the abundance of CXCR2$^+$ neutrophils in the peripheral blood of BALB/c mice at T0 (left), T6 (middle), and T11 (right). **B**. Representative dot-plots of CD124$^+$ neutrophils in peripheral blood of BALB/c (upper row) and CD-1 (lower row) mice at T0 (left), T6 (middle), and T11 (right).

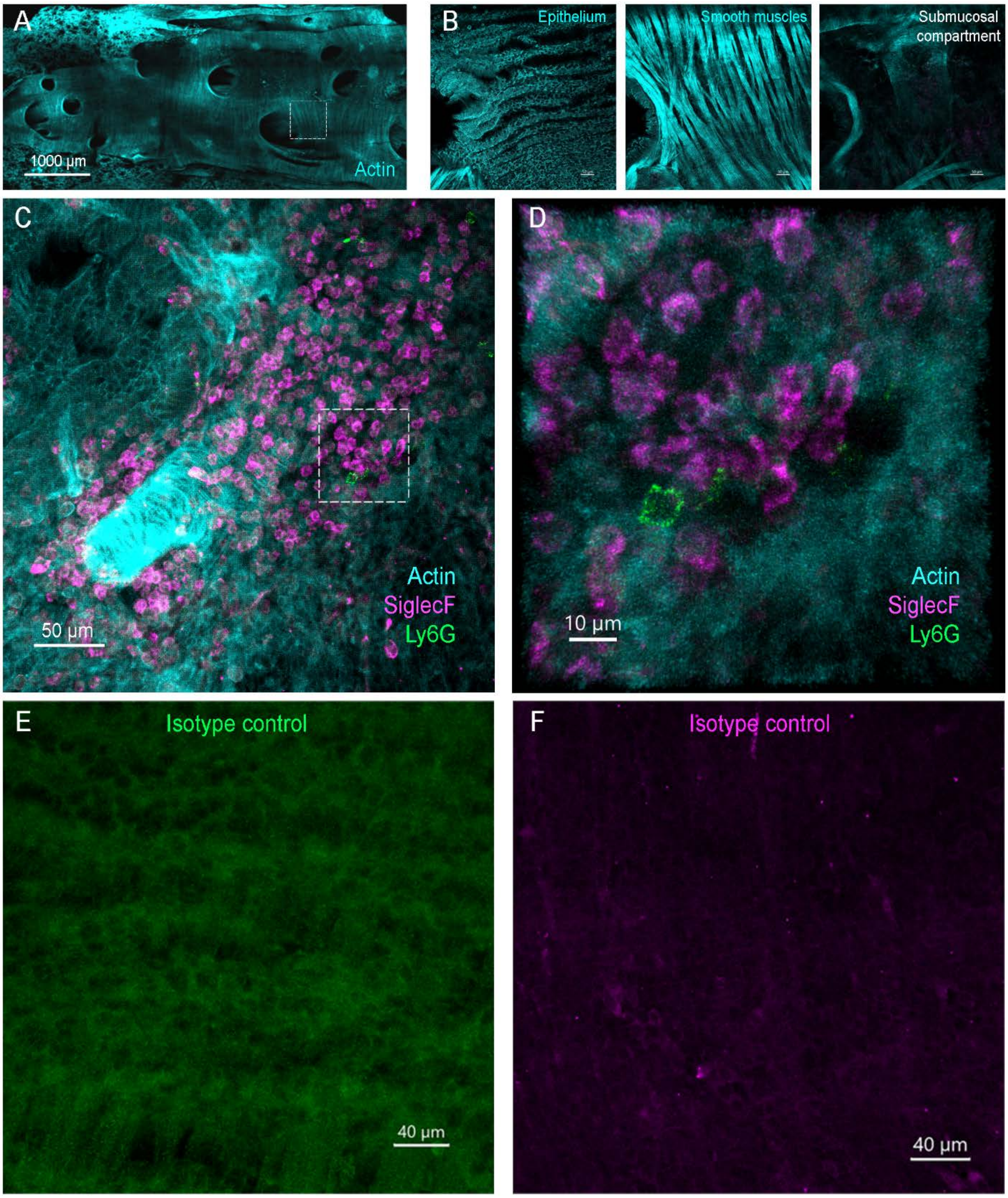

Supplementary Figure 3. Eosinophils and neutrophils in the lung tissue. A. Representative image of conducting airway (main bronchus) of a mouse stained with phalloidin (Actin, cyan) as a whole-mount. Scale bar 1000 µm. B. Enlarged region indicated in A shown as Z-sections of the epithelial layer (left), smooth muscle layer (middle), and submucosal compartment (right). Scale bar 50 µm. C. Representative image of conducting airway specimen of a mouse with allergic airway inflammation at T6 showing actin-rich epithelial and smooth muscle cells (Actin, cyan), eosinophils (SiglecF, magenta), and neutrophils (Ly6G, green). Scale bar 50 µm. D. Enlarged region boxed in C demonstrated a few neutrophils (green) and many eosinophils (magenta) in the lung parenchyma. Scale bar 10 µm. E, F. Respective isotype controls. Scale bar 40 µm.

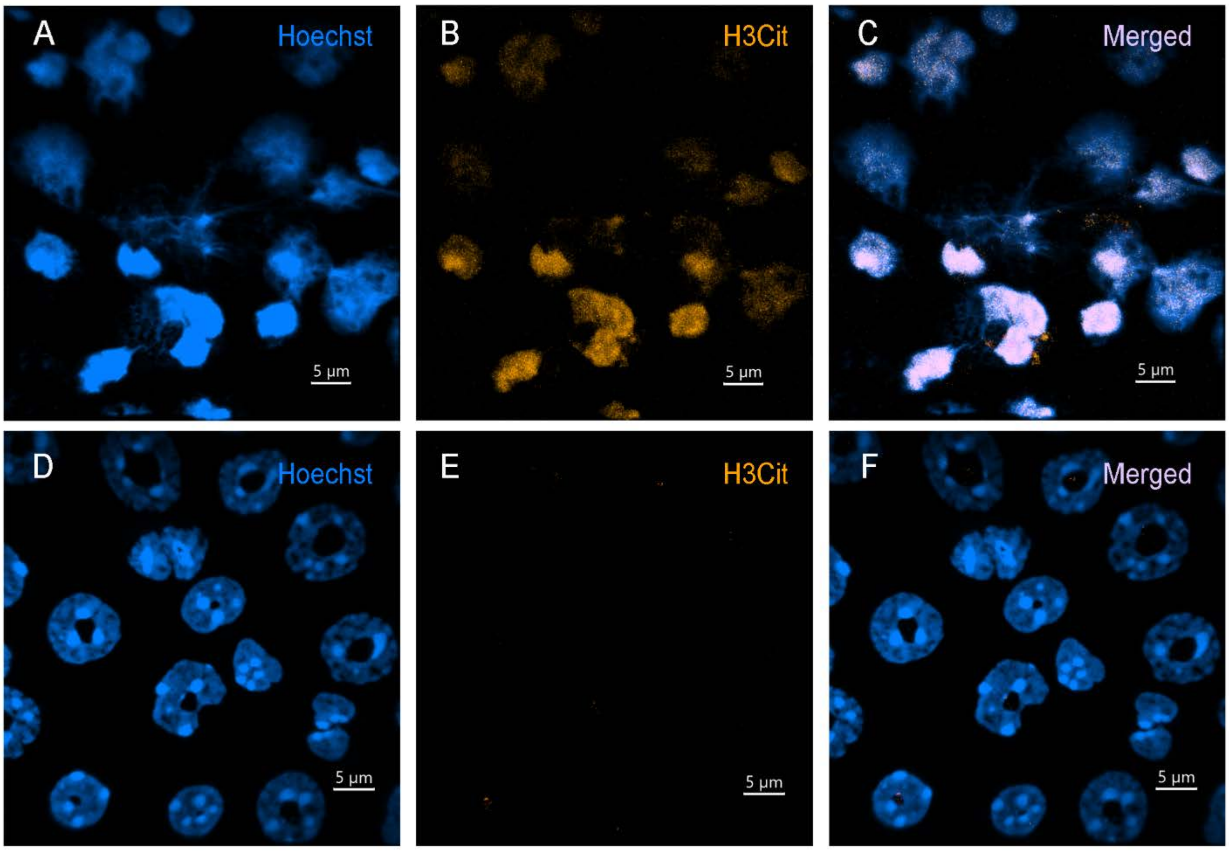

Supplementary Figure 4. Imaging of NET formation or necrosis. The representative image of neutrophils yielded from bone marrow of mice with allergic airway inflammation. A-F. Representative images of neutrophils incubated in the presence of PMA (A-C) or without PMA (D-F). Nuclei were stained by Hoechst 33342 (A, D); for netosis detection antibodies for citrullinated histone H3 were used (B, E); merged images (C, F).

Supplemetary Table 1. Mouse BAL Th2 cytokines.

Assay sensitivity (pg/mL):		2.2	2.7	2.3	1.7	2.5	2.3
**sample**	**dilution**	**IL-5 (A5) pg/ml**	**TNF-α (A6) pg/ml**	**IL-6 (A8) pg/ml**	**IL-4 (A10) pg/ml**	**IL-10 (B2) pg/ml**	**IL-13 (B9) pg/ml**
BALBc-Af-40ug-1-1	1	nd	0	nd	0	nd	nd
BALBc-Af-40ug-1-2	1	nd	0	nd	nd	nd	nd
BALBc-Af-40ug-2-1	1	nd	84.12	5.49	nd	nd	nd
BALBc-Af-40ug-2-2	1	nd	97.24	6.31	nd	nd	nd
BALBc-Af-40ug-3-1	1	nd	0	nd	nd	nd	nd
BALBc-Af-40ug-3-2	1	nd	0	nd	nd	nd	nd
BALBc-Af-40ug-4-1	1	nd	0	nd	0	nd	nd
BALBc-Af-40ug-4-2	1	nd	0	nd	nd	nd	nd
BALBc-Af-4ug-T6-1-1	1	0	0	nd	0	nd	nd
BALBc-Af-4ug-T6-1-2	1	nd	0	nd	0	nd	nd
BALBc-Af-4ug-T6-2-1	1	3.27	9.24	3.16	0	nd	nd
BALBc-Af-4ug-T6-2-2	1	4.20	12.89	3.10	0	nd	nd
BALBc-Af-4ug-T6-3-1	1	nd	0	nd	0	nd	nd
BALBc-Af-4ug-T6-3-2	1	nd	0	nd	0	nd	nd
BALBc-Af-4ug-T6-4-1	1	6.58	10.55	3.73	2.59	64.96	2.52
BALBc-Af-4ug-T6-4-2	1	5.32	8.75	nd	0	32.15	0
BALBc-Af-4ug-T11-1-1	1	nd	0	nd	nd	nd	nd
BALBc-Af-4ug-T11-1-2	1	0	0	nd	nd	nd	nd
BALBc-Af-4ug-T11-2-1	1	nd	nd	nd	nd	nd	nd
BALBc-Af-4ug-T11-2-2	1	nd	nd	nd	nd	nd	nd
BALBc-Af-4ug-T11-3-1	1	nd	0	nd	1.91	nd	nd
BALBc-Af-4ug-T11-3-2	1	nd	0	nd	1.92	nd	nd
BALBc-Af-4ug-T11-4-1	1	nd	0	nd	0	nd	nd
BALBc-Af-4ug-T11-4-2	1	nd	0	nd	0	nd	nd
BALBc-NM-1-1	1	0	0	0	0	nd	nd
BALBc-NM-1-2	1	0	0	0	0	nd	nd
BALBc-NM-2-1	1	nd	nd	nd	0	nd	nd
BALBc-NM-2-2	1	0	nd	nd	nd	nd	nd
BALBc-NM-3-1	1	nd	0	nd	nd	nd	nd
BALBc-NM-3-2	1	nd	0	nd	nd	nd	nd
BALBc-NM-4-1	1	nd	0	nd	nd	nd	nd
BALBc-NM-4-2	1	nd	0	nd	0	nd	nd
CD-Af-40ug-1-1	1	nd	8.22	0	nd	nd	nd
CD-Af-40ug-1-2	1	nd	4.54	0	nd	nd	nd
CD-Af-40ug-2-1	1	0	49.96	7.54	0	56.07	0
CD-Af-40ug-2-2	1	nd	2.91	0	nd	nd	nd
CD-Af-40ug-3-1	1	nd	nd	nd	nd	nd	nd
CD-Af-40ug-3-2	1	nd	0	nd	nd	nd	nd
CD-Af-40ug-4-1	1	nd	11.63	2.34	nd	nd	nd
CD-Af-40ug-4-2	1	nd	6.7	0	nd	nd	nd
CD1-Af-4ug-T6-1-1	1	nd	0	nd	0	nd	nd
CD1-Af-4ug-T6-1-2	1	nd	0	nd	0	nd	nd
CD1-Af-4ug-T6-2-1	1	nd	0	nd	0	nd	nd
CD1-Af-4ug-T6-2-2	1	nd	0	nd	nd	nd	nd
CD1-Af-4ug-T6-3-1	1	nd	0	nd	nd	nd	nd
CD1-Af-4ug-T6-3-2	1	nd	0	nd	0	nd	nd
CD1-Af-4ug-T6-4-1	1	nd	0	nd	nd	nd	nd
CD1-Af-4ug-T6-4-2	1	nd	0	nd	nd	nd	nd
CD1-Af-4ug-T11-1-1	1	0	10.89	2.48	2.05	65.93	2.31
CD1-Af-4ug-T11-1-2	1	0	10.50	2.77	2.93	66.97	0
CD1-Af-4ug-T11-2-1	1	nd	0	nd	0	nd	nd
CD1-Af-4ug-T11-2-2	1	nd	0	nd	0	nd	nd
CD1-Af-4ug-T11-3-1	1	nd	4.85	7.62	2.34	nd	0
CD1-Af-4ug-T11-3-2	1	nd	2.85	0	nd	nd	nd
CD1-Af-4ug-T11-4-1	1	nd	0	nd	nd	nd	nd
CD1-Af-4ug-T11-4-2	1	nd	0	nd	0	nd	nd
CD1-NM-1-1	1	nd	0	nd	0	nd	nd
CD1-NM-1-2	1	nd	nd	nd	nd	nd	nd
CD1-NM-2-1	1	nd	nd	nd	nd	nd	nd
CD1-NM-2-2	1	nd	0	nd	nd	nd	nd
CD1-NM-3-1	1	0	nd	nd	nd	nd	nd
CD1-NM-3-2	1	nd	nd	nd	0	nd	nd
CD1-NM-4-1	1	nd	nd	nd	nd	nd	nd
CD1-NM-4-2	1	nd	nd	nd	nd	nd	nd

Th2 cytokine concentrations (pg / mL) in BAL of intact mice (NM), and mice at different time points (T6, T11) of allergic airway inflammation (4 μg / mouse) and in mice with acute inflammation (40 μg / mouse).

Supplementary Table 2. Mouse BAL cytokines.

sample	dilution	CXCL1 (KC) (A4) pg/ml	IL-18 (A6) pg/ml	IL-23 (A7) pg/ml	IL-12p70 (B2) pg/ml	IL-6 (B3) pg/ml	TNF-α (B4) pg/ml	IL-12p40 (B7) pg/ml	IL-1β (B9) pg/ml
Assay sensitivity (pg/mL):		2	7.7	3.3	1.1	2.0	2.4	1.6	2.3
Seria I									
CD1-Af-4ug-T6-1-1	1	72.03	nd	nd	nd	nd	nd	44.33	nd
CD1-Af-4ug-T6-1-2	1	64.86	nd	nd	nd	nd	nd	37.72	nd
CD1-Af-4ug-T6-2-1	1	7.86	nd	nd	nd	nd	nd	4.51	nd
CD1-Af-4ug-T6-2-2	1	7.09	nd	nd	nd	nd	nd	4.90	nd
CD1-Af-4ug-T6-3-1	1	3.62	nd	nd	nd	nd	nd	2.62	nd
CD1-Af-4ug-T6-3-2	1	3.64	nd	nd	nd	nd	nd	2.73	nd
CD1-Af-4ug-T6-4-1	1	0	nd	nd	nd	nd	nd	0	nd
CD1-Af-4ug-T6-4-2	1	0	nd	nd	nd	nd	nd	0	nd
CD1-NM-1-1	1	0	nd	nd	nd	nd	nd	0	nd
CD1-NM-1-2	1	0	nd	nd	nd	nd	nd	0	nd
CD1-NM-2-1	1	0	nd	nd	nd	nd	nd	0	nd
CD1-NM-2-2	1	nd	nd	nd	nd	nd	nd	nd	nd
Seria II									
CD1-Af-4ug-T6-1-1	1	11.84	nd	nd	nd	nd	nd	5.69	nd
CD1-Af-4ug-T6-1-2	1	11.66	nd	nd	nd	nd	nd	4.54	nd
CD1-Af-4ug-T6-2-1	1	50.81	nd	nd	nd	nd	nd	28.88	nd
CD1-Af-4ug-T6-2-2	1	49.65	nd	nd	nd	nd	nd	27.86	nd
CD1-Af-4ug-T6-3-1	1	4.84	nd	nd	nd	nd	nd	3.56	nd
CD1-Af-4ug-T6-3-2	1	5.46	nd	nd	nd	nd	nd	4.15	nd
CD1-Af-4ug-T6-4-1	1	14.76	nd	nd	nd	nd	nd	13.83	nd
CD1-Af-4ug-T6-4-2	1	16.54	nd	nd	nd	nd	nd	15.76	nd
CD1-Af-4ug-T11-1-1	1	32.63	nd	nd	nd	nd	7.22	20.10	nd
CD1-Af-4ug-T11-1-2	1	31.08	nd	nd	nd	nd	6.54	19.57	nd
CD1-Af-4ug-T11-2-1	1	18.43	nd	nd	nd	nd	nd	8.60	nd
CD1-Af-4ug-T11-2-2	1	18.38	nd	nd	nd	nd	nd	7.76	nd
CD1-Af-4ug-T11-3-1	1	31.88	nd	nd	nd	nd	nd	20.09	nd
CD1-Af-4ug-T11-3-2	1	33.77	nd	nd	nd	nd	nd	20.84	nd
CD1-Af-4ug-T11-4-1	1	89.05	nd	nd	nd	nd	nd	14.74	nd
CD1-Af-4ug-T11-4-2	1	85.53	nd	nd	nd	nd	nd	14.68	nd
CD1-NM-1-1	1	11.96	nd	nd	nd	nd	nd	0	nd
CD1-NM-1-2	1	11.62	nd	nd	nd	nd	nd	0	nd
CD1-NM-2-1	1	6.63	nd	nd	nd	nd	nd	2.08	nd
CD1-NM-2-2	1	7.15	nd	nd	nd	nd	nd	2.44	nd
CD1-NM-3-1	1	8.71	nd	nd	nd	nd	nd	2.11	nd
CD1-NM-3-2	1	8.19	nd	nd	nd	nd	nd	2.19	nd
CD1-NM-4-1	1	13.32	nd	nd	nd	nd	nd	5.05	nd
CD1-NM-4-2	1	15.63	nd	nd	nd	nd	nd	6.77	nd
Seria III									
CD1-Af-40ug-1-1	1	14.02	nd	nd	nd	nd	nd	112.75	nd
CD1-Af-40ug-1-2	1	13.21	nd	nd	nd	nd	nd	122.13	nd
CD1-Af-40ug-2-1	1	11.15	nd	nd	nd	nd	nd	70.29	nd
CD1-Af-40ug-2-2	1	11.45	nd	nd	nd	nd	nd	81.66	nd
CD1-Af-40ug-3-1	1	0	nd	nd	nd	nd	nd	0	nd
CD1-Af-40ug-3-2	1	0	nd	nd	nd	nd	nd	0	nd
CD1-Af-40ug-4-1	1	40.11	nd	nd	nd	nd	2.48	110.93	nd
CD1-Af-40ug-4-2	1	41.33	nd	nd	nd	nd	2.55	117.59	nd
CD1-Af-40ug-5-1	1	15.45	nd	nd	nd	nd	nd	173.06	nd
CD1-Af-40ug-5-2	1	12.01	nd	nd	nd	nd	nd	96.42	nd
CD1-Af-40ug-6-1	1	14.26	nd	nd	nd	nd	nd	172.22	nd
CD1-Af-40ug-6-2	1	12.43	nd	nd	nd	nd	nd	110.03	nd
CD1-Af-4ug-T6-1-1	1	4.92	nd	nd	nd	nd	nd	1.69	nd
CD1-Af-4ug-T6-1-2	1	5.26	nd	nd	nd	nd	nd	1.79	nd
CD1-Af-4ug-T6-2-1	1	28.65	nd	nd	nd	nd	nd	13.58	nd
CD1-Af-4ug-T6-2-2	1	27.13	nd	nd	nd	nd	nd	13.20	nd
CD1-Af-4ug-T6-3-1	1	2.08	nd	nd	nd	nd	nd	0	nd
CD1-Af-4ug-T6-3-2	1	2.13	nd	nd	nd	nd	nd	1.66	nd
CD1-Af-4ug-T6-4-1	1	5.71	nd	nd	nd	nd	nd	4.80	nd
CD1-Af-4ug-T6-4-2	1	5.99	nd	nd	nd	nd	nd	5.20	nd
CD1-Af-4ug-T11-1-1	1	15.18	nd	nd	nd	nd	3.31	6.56	nd
CD1-Af-4ug-T11-1-2	1	17.10	nd	nd	nd	nd	3.31	5.70	nd
CD1-Af-4ug-T11-2-1	1	8.60	nd	nd	nd	nd	0	2.59	nd
CD1-Af-4ug-T11-2-2	1	8.40	nd	nd	nd	nd	0	2.27	nd
CD1-Af-4ug-T11-3-1	1	18.00	nd	nd	nd	nd	nd	7.19	nd
CD1-Af-4ug-T11-3-2	1	17.98	nd	nd	nd	nd	nd	6.63	nd
CD1-Af-4ug-T11-4-1	1	55.56	nd	nd	nd	nd	0	3.49	nd
CD1-Af-4ug-T11-4-2	1	62.37	nd	nd	nd	nd	2.80	3.58	nd
CD1-NM-1-1	1	4.77	nd	nd	nd	nd	nd	0	nd
CD1-NM-1-2	1	4.58	nd	nd	nd	nd	nd	0	nd
CD1-NM-2-1	1	2.30	nd	nd	nd	nd	nd	0	nd
CD1-NM-2-2	1	2.57	nd	nd	nd	nd	nd	0	nd
CD1-NM-3-1	1	3.80	nd	nd	nd	nd	nd	0	nd
CD1-NM-3-2	1	3.71	nd	nd	nd	nd	nd	0	nd
CD1-NM-4-1	1	4.73	nd	nd	nd	nd	nd	0	nd
CD1-NM-4-2	1	3.57	nd	nd	nd	nd	nd	0	nd

Cytokine concentrations (pg / mL) in BAL of intact mice (NM), and mice at different time points (T6, T11) of allergic airway inflammation (4 µg / mouse) and in mice with acute inflammation (40 µg / mouse).

Supplementary Table 3. Mouse Th BAL cytokines.

Assay sensitivity (pg/mL):		2,3	2,2	2,7	1,7	1,7	1,8	2,5	2,2	2,1	2	2	2,3
**sample**	**dilution**	**IFN-γ (A4)**	**IL-5 (A5)**	**TNF-α (A6)**	**IL-2 (A7)**	**IL-6 (A8)**	**IL-4 (A10)**	**IL-10 (B2)**	**IL-9 (B3)**	**IL-17A (B4)**	**IL-17F (B5)**	**IL-22 (B7)**	**IL-13 (B9)**
CD1-Af-4ug-T6-1-1	1	nd	nd	nd	nd	nd	nd	nd	nd	nd	nd	nd	nd
CD1-Af-4ug-T6-1-2	1	nd	nd	72.65	nd	nd	nd	nd	nd	nd	nd	nd	nd
CD1-Af-4ug-T6-2-1	1	nd	nd	nd	nd	nd	0	nd	nd	nd	nd	nd	nd
CD1-Af-4ug-T6-2-2	1	nd	nd	nd	nd	nd	nd	nd	nd	nd	nd	nd	nd
CD1-Af-4ug-T6-3-1	1	0	nd	nd	nd	nd	nd	nd	nd	nd	nd	nd	nd
CD1-Af-4ug-T6-3-2	1	0	nd	nd	nd	3.14	nd	5.39	nd	nd	nd	nd	nd
CD1-Af-4ug-T6-4-1	1	0	nd	nd	nd	nd	1.8	nd	nd	nd	nd	nd	nd
CD1-Af-4ug-T6-4-2	1	0	nd	nd	nd	nd	nd	nd	nd	nd	nd	nd	nd
CD1-Af-4ug-T11-1-1	1	0	nd	nd	0	nd	1.84	0	nd	nd	nd	nd	nd
CD1-Af-4ug-T11-2-1	1	0	nd	nd	3.9	4.81	nd	nd	nd	nd	nd	nd	nd
CD1-Af-4ug-T11-3-1	1	0	nd	nd	0	nd	nd	nd	nd	nd	nd	nd	nd
CD1-Af-4ug-T11-4-1	1	nd	nd	53.64	nd	nd	nd	nd	nd	nd	nd	nd	nd
CD1-Af-40ug-1-1	1	nd	17.89	nd	0	nd	0	nd	nd	nd	nd	nd	nd
CD1-Af-40ug-2-1	1	0	0	nd	3.59	nd	nd	nd	nd	nd	nd	nd	nd
CD1-Af-40ug-3-1	1	nd	nd	nd	2.3	nd	nd	nd	nd	nd	nd	nd	nd
CD1-Af-40ug-4-1	1	2.6	20.66	23.53	0	nd	nd	nd	nd	nd	nd	nd	nd
CD1-NM-1-1	1	0	nd	0	0	nd	nd	nd	nd	nd	nd	nd	nd
CD1-NM-1-2	1	0	8.34	nd	4.3	nd	nd	nd	nd	nd	nd	nd	nd
CD1-NM-2-1	1	0	nd	nd	1.72	nd	nd	nd	nd	nd	nd	nd	nd
CD1-NM-2-2	1	nd	nd	nd	nd	nd	0	nd	nd	nd	nd	nd	nd
CD1-NM-3-1	1	0	nd	nd	nd	nd	nd	nd	nd	nd	nd	nd	nd
CD1-NM-3-2	1	nd	nd	nd	nd	nd	nd	nd	nd	nd	nd	nd	nd

Mouse Th cytokine concentrations (pg / mL) in BAL of intact mice (NM), and mice at different time points (T6, T11) of allergic airway inflammation (4 μg / mouse) and in mice with acute inflammation (40 μg / mouse).
